# Non-invasive indicators associated with the milk yield response after anthelmintic treatment at calving in dairy cows

**DOI:** 10.1186/s12917-014-0264-x

**Published:** 2014-11-14

**Authors:** Sien H Verschave, Jozef Vercruysse, Andrew Forbes, Geert Opsomer, Miel Hostens, Luc Duchateau, Johannes Charlier

**Affiliations:** Department of Virology, Parasitology and Immunology, Faculty of Veterinary Medicine, Ghent University, Salisburylaan 133, 9820 Merelbeke, Belgium; Merial SAS, 29 Avenue Tony Garnier, Lyon, 69007 France; Department of Reproduction, Obstetrics and Herd Health, Faculty of Veterinary Medicine, Ghent University, Salisburylaan 133, 9820 Merelbeke, Belgium; Department of Physiology and Biometrics, Faculty of Veterinary Medicine, Ghent University, Salisburylaan 133, 9820 Merelbeke, Belgium

**Keywords:** Dairy cattle, Gastrointestinal nematodes, Targeted selective treatment, Anti-*O. ostertagi* antibody level, Faecal egg counts, Eprinomectin

## Abstract

**Background:**

Gastrointestinal nematodes are an important cause of reduced performance in cattle. Previous studies in Europe showed that after anthelmintic treatment an average gain in milk production of around 1 kg per day/cow can be expected. However, (1) these studies have mainly evaluated group-based anthelmintic treatments during the grazing season or at housing and (2) little is known about parameters affecting variations in the treatment response amongst cows. A better knowledge of such parameters could help to select animals that benefit most from treatment and thus lead to a more rational use of anthelmintics. Therefore, a randomized, non-blinded, controlled clinical trial was performed on 11 commercial dairy farms (477 animals) in Belgium, aiming (1) to study the effect of eprinomectin treatment at calving on milk production and (2) to investigate whether the milk yield response was related to non-invasive animal parameters such that these could be used to inform targeted selective treatment decisions.

**Results:**

Analyses show that eprinomectin treatment around calving resulted in an average (± standard error) increase of 0.97 (±0.41) kg in daily milk yield that was followed up over 274 days on average. Milk yield responses were higher in multiparous compared to primiparous cows and in cows with a high (4^th^ quartile) anti-*O. ostertagi* antibody level in a milk sample from the previous lactation. Nonetheless, high responses were also seen in animals with a low (1^st^ quartile) anti-*O. ostertagi* antibody level. In addition, positive treatment responses were associated with higher faecal egg counts and a moderate body condition score at calving (2^nd^ quartile).

**Conclusions:**

In conclusion, this study provides novel insights into the production response after anthelmintic treatment at calving and factors which influence this. The data could be used to support the development of evidence-based targeted selective anthelmintic treatment strategies in dairy cattle.

## Background

Gastrointestinal nematodes (GI) are an important cause of reduced performance in grazing cattle. A review of studies on the effect of anthelmintic treatment on milk production from 2000 onwards reported an average milk yield response of 1 kg/cow per day [1]. At present, two innovative concepts are described to prevent nematode-associated production losses while preserving anthelmintic drug efficacy, namely targeted treatment (TT) and target selective treatment (TST) [2]. Using TT, the whole group or herd is treated at an optimal time based on parameters that quantify the risk of infection and/or production losses. A useful parameter currently available for TT in adult dairy cattle is the anti-*O. ostertagi* antibody level in milk [3]. When applying TST, only selected individuals are treated, the aim being to lower the risk for the development of anthelmintic resistance by increasing the size of the parasite population in *refugia*. However, practical implementation of TST at the farm level is still limited by the lack of data on which indicators are useful to identify animals that would benefit most from treatment. In Europe, all recent clinical studies, evaluating the effect of anthelmintic treatment on milk production can be considered as TT, as they target whole herds, and have been restricted to mid-season or housing treatments [4-8]. However, a cost-benefit analysis suggested that the economic benefit of anthelmintic treatment of dairy cows is considerably larger when cows are treated around calving compared to treatment at housing [9]. Studies assessing the effect of anthelmintic treatment around calving have been carried out in Canada [10-12] and New Zealand [13], but significant differences in climate and farm management practices may result in different parasite epidemiology and different production effects when compared to European circumstances.

Most clinical trials evaluating the effect of anthelmintic treatment, report the average effect in the study population. It is well recognized that treatment responses can vary largely within and amongst herds [14] and depend on factors such as infection level and management [15]. Less is known about easy-to-use animal parameters that are potentially related to the within-herd variation of production responses, or that could act as a proxy for such responses. A better understanding of these relationships is key to identifying the animals that would benefit most from anthelmintic drug administration, consistent with the TST approach. Previously some indicators have been associated with the milk yield response after anthelmintic treatment. The parameter yielding most consistent results is the anti-*Ostertagia ostertagi* antibody level in individual milk samples, with studies showing higher treatment responses in cows with a high antibody level [3,11,12,16]. As for the age of the cow, some studies found no relationship with treatment effect [10,14,17], whereas others reported a higher milk yield gain in older cows [3,13] or conversely, in younger cows [5]. Finally, it was suggested that treatment responses were higher in high producing animals [14,18], but this was not observed by others [10].

A problem associated with previous studies is that mostly the study design was aimed at investigating the overall treatment effect, leading to low statistical power or unbalanced data for evaluating the effect of indicators associated with the treatment response. Therefore, we performed a randomized, non-blinded, controlled clinical trial with herd (< vs. > average herd production level), *O. ostertagi*-antibody levels in individual milk samples (optical density ratio (ODR) < vs. ≥0.5) and age (2^nd^ vs. ≥3^rd^ lactation) as blocking factors for treatment assignment. The aim were (1) to investigate the effect of anthelmintic treatment at calving on milk production in dairy cattle and (2) to evaluate if some easy-to-use animal parameters (i.e. parity, body condition score, pre-treatment anti-*O. ostertagi* antibody levels) are associated with the milk production response following anthelmintic treatment.

## Methods

Following the recommendations of Belgian and European legislation (KB 11/5/2007; 86/609/EEC), this field study conducted on commercial dairy herds did not require ethical approval.

### Selection of farms

The study was conducted on 11 dairy herds located in Flanders, Belgium. On average 73 lactating animals, mainly Holstein Friesians, were present on these farms, while the rolling herd average ranged between 7262 and 10920 kg milk. Cows calved all year round. The following criteria were applied for the selection of farms: (1) cows had previous access to pasture and were naturally infected with gastrointestinal nematodes (i.e. anti-*O. ostertagi* ELISA results on bulk milk samples of April 2011 ≥ 0.6 ODR), (2) the last treatment of the cows against GI nematodes had been performed ≥6 months before the experimental treatment and (3) participation in the milk production registration program of CRV (Arnhem, The Netherlands) to enable standardized data collection.

### Study design

A non-blinded, randomized, controlled clinical trial was performed to evaluate the effect of anthelmintic treatment at calving on milk production and to evaluate the association with easy-to-use indicators. Both first-calving heifers and older animals were included in the trial. Within 15 days after calving the animals either received treatment with eprinomectin (Eprinex® pour-on, Merial) at a dosage of 0.1 ml per kg bodyweight (500 μg/kg) or received no treatment. Treatments were performed by the farmer, based on the estimated bodyweight. The capability of the participating farmers to estimate the body weight of their animals was tested during farm visits on which the body weight of several random animals was measured based on the heart girth. These measurements revealed that when body weights were underestimated the underestimations were generally low (<50 kg).

The multiparous cows were randomly assigned within herd using *O. ostertagi* antibody level in individual milk sample measured in April 2011 (< vs. ≥0.5 ODR), cow parity (2^nd^ vs. ≥3^rd^ lactation) and previous production level (< vs. > herd-average) as blocking factors. The treatment assignments were communicated to the farmer through a hard-copy attention list, generated with the aid of the herd management software (Veemanager, CRV, Arnhem, The Netherlands) and specifying cow identification, expected calving date and the randomly assigned group (treatment or no treatment).

Due to the lack of an ODR measurement from the previous lactation, heifers could not be included in the randomized list. Therefore, the farmer was asked to alternate treatment assignments (yes/no) for this group of animals following the order in which they calved.

The farmer was requested to complete the hard-copy attention list at the time of treatment by noting the body condition score (BCS; according to [19]), the estimated body weight, dosage applied and the date of treatment. A scale from 1 to 5 was used for the body condition scoring, where 1 stands for severe under-conditioned and 5 for severe over-conditioned. At the start of the trial all farmers received a body condition scoring chart based on Edmonson et al. [19], as a visual aid.

It is acknowledged that the use of anthelmintic pour-on treatments in individual cattle potentially compromises the precision of delivery of the recommended dosage to the intended animal and may result in sub-optimal dosing to in-contact animals too [20]. Therefore, farmers were advised to prevent close contact between treated and untreated animals in the first hours following treatment. Most did this by treating animals in a separate calving box several hours before allowing them to rejoin the lactating animals. This is an important logistical consideration if TST is to be practised on commercial farms, because this is an inevitable compromise as it is generally impractical to keep recently treated and untreated cattle apart for adequate periods.

All concomitant anthelmintic and ectoparasiticide treatments, other than those defined in the study protocol were documented, so that these animals could be excluded from the analysis. Cattle were enrolled from July 2011 until September 2012. The progress and compliance with the study protocol was monitored through monthly telephone contacts and three farm visits during the study period.

### Collection of faecal samples and faecal egg counts

On two of the 11 dairy herds, the farmers took faecal samples at the moment of treatment and these were stored in a refrigerator until collection on a weekly basis. Samples were processed immediately after arrival at the lab and examined for nematode eggs by the FLOTAC® method based on the manufacturer’s instructions. A saturated sucrose-salt solution was used as flotation solution (density 1.27) and the suspension was distributed over one chamber (1 × 5 ml), resulting in an analytic sensitivity of 2 eggs per gram faeces (EPG; [21]).

### Collection of milk samples and *O. ostertagi* milk ELISA

Bulk tank milk samples were collected at monthly intervals from July 2011 until July 2012. For logistical reasons, bulk milk data for the month of April 2012 were not available. Individual milk samples from all lactating animals were collected at three-monthly intervals starting from July 2011 until July 2012 as part of routine sampling for quality control and milk production registration, in cooperation with the Milk Control Centre, Flanders (MCC, Lier, Belgium) and CRV (Arnhem, The Netherlands).

The collected milk samples were subjected to a commercially available antibody-detection *O. ostertagi* ELISA (SVANOVIR®*O. ostertagi*-Ab, Boehringer Ingelheim Svanova, Uppsala) according to the manufacturer’s instructions, at the laboratories of the MCC. The results were expressed as an optical density ratio (ODR) that is calculated following the formula ODR = (OD - NC)/(PC - NC), where OD is the result of the optical density reading of the sample at 405 nm, and NC and PC are the OD of the negative and positive control samples, respectively.

### Collection of milk production data and processing

Individual milk production records were collected from the CRV milk recording program, with a 4 to 6 week sampling interval; the parameters used were: kg milk, somatic cell count (SCC)/1000, breed, days in milk and lactation number. The milk production records were also subjected to the MilkBot® lactation model (DairySight LLC, Argyle, New York) to create a lactation curve [22]. Both the shape and magnitude of the lactation curve are quantified by the model as a set of parameter values, each of which is associated with a single aspect of lactation curve shape. Analysis of the Milkbot® parameters allows the detection of changes in the distribution of milk production that are not apparent when only daily milk weights or totals are analyzed. The parameter “scale” is a measure of magnitude, without changing the shape of the curve. The parameter “ramp” measures the steepness of the post-parturient rise in production. The parameter “decay” is used to measure the rate of decline in production after the peak in milk production.

### Statistical data-analysis

The effect of eprinomectin treatment on the anti-*O. ostertagi* antibody levels was analysed through a linear mixed model with herd and cow nested in herd as random effects. Treatment (yes/no), days after treatment, the month at which the milk was tested and an interaction term between treatment and days after treatment were used as fixed effects in the model. Because the herds were sampled at 3-monthly intervals, the variable ‘days after treatment’ was categorized in 3 intervals: ‘0–3 months’, ‘3–6 months’, ‘>6 months’. To investigate the correlation between the faecal egg counts, measured around the moment of calving, and the anti-*O. ostertagi* antibody level, measured in individual milk samples within 30 days post calving, the Spearman rank correlation coefficient was used.

The effect of eprinomectin treatment on milk production parameters was first analysed through a linear mixed model with the test day milk production (kg milk) as outcome variable and herd and cow nested in herd as random effects. Treatment (yes/no), lactation number (‘1^st^’, ‘2^nd^’, ‘3^rd^ or higher’), the natural logarithm of SCC/1000, number of days in milk (dim), wilmink’s function (dim^-0.05^) and year quarter in which calving occurred, were introduced as fixed effects in the model.

In order to investigate between-herd variation in treatment responses, the analysis was repeated for each herd separately. Results were presented by a forest plot and an overall treatment effect was obtained by the precision weighted average, using a random effects model with herd as the random effect.

The relationship between the indicators and the milk production response after treatment was evaluated using the model mentioned above. Linear interaction terms were evaluated between the indicators and treatment effect. These interaction terms were not significant (α =0.05), except for faecal egg counts. Non-linear effects were explored by categorizing the data. Continuous parameters (anti-*O. ostertagi* antibody level and BCS centered to the herd mean) were categorized according to their quartiles. The moment of treatment was categorized in housing (November until March) and pasture period (April until October) based on Bennema et al. [23]. When several pre-treatment *O. ostertagi* ELISA results for a cow were available, the result of the sample closest to calving was used. The treatment effect was estimated within each category of the pre-treatment anti-*O. ostertagi* antibody level, lactation number, the BCS centered to the herd mean and season (pasture vs. housed) in which treatment occurred.

To evaluate heteroscedasticity and the normality of the residuals, plots of the residuals and predicted values were performed. All analyses were carried out with the PROC MIXED or PROC CORR command in the software package SAS version 9.3 (SAS institute Inc., Cary, NC, USA), except for the forest plot and precision weighted treatment effect, which was computed by the ‘metafor’ package in R (Cran).

## Results

### Herd characteristics and treatment allocation

Analysis of the effect of treatment on the anti-*O. ostertagi* antibody levels was based on data obtained from 498 animals present in the participating herds. The data used to analyse the effect of treatment on milk production, were based on 477 cows from all participating herds. Of these, 234 belonged to the treatment group and 243 to the control group. The treatment response on the test day milk production records was followed up during 274 days of lactation on average. Table 1 shows that the average anti-*O. ostertagi* antibody level of individual milk samples before treatment of both groups, lactation number, breed and the year quarter in which calving occurred were evenly distributed between the treated and untreated animals indicating a successful treatment allocation. Concomitant treatments were recorded on 3 of the participating herds, for a total of 39 animals. All these treatments were related to liver fluke infections and the anthelmintics used were either closantel or oxyclozanide, neither of which has any activity against *O. ostertagi*.

**Table 1:** **Number of cows, average ± standard deviation of anti-** ***O. ostertagi*** **antibody levels in individual milk before treatment registration and distribution of breed, lactation number and year quarter of calving in the treated and untreated group**

**Parameter**	**Eprinomectin**	**Control**
**Number of cows**	234	243
**Anti-** ***O. ostertagi*** **antibody level (ODR)**	0.49 ± 0.3	0.52 ± 0.3
**Breed (%):**		
Holstein Friesian	43	46
Other	6	5
**Lactation number (%):**		
First	14	12
Second	13	13
Third or higher	26	22
**Year quarter:**		
Jan - Mar	10	14
Apr - Jun	13	8
Jul - Sep	13	12
Oct - Dec	15	15

### Parasitological parameters

**Figure 1:**
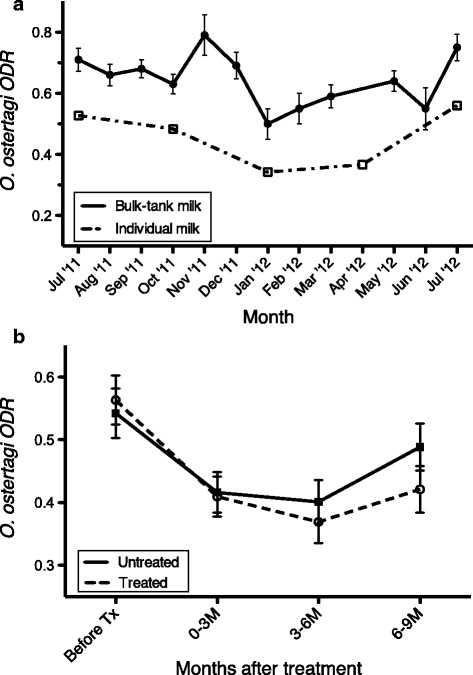
**Anti-O. ostertagi antibody levels (ODR) in bulk-tank and individual milk samples during the study period. (a)** Course of the anti-*O. ostertagi* antibody levels (ODR) during the study period in bulk-tank and individual milk samples on the 11 herds. **(b)** The course of the anti-*O. ostertagi* antibody levels (ODR) relative to the month of calving in individual milk samples of 1274 cows coming from all the 11 herds. Bars represent standard error of the mean.

Figure 1a shows the course of the anti-*O. ostertagi* antibody levels recorded in bulk-tank and individual milk samples during the study period. Figure 1b shows the course of the anti-*O. ostertagi* antibody levels in individual milk samples relative to the month of treatment. In both the treated and untreated group, antibody levels decreased after calving. However, there was a significant interaction (*P* = 0.02) between treatment and the time variable “month after treatment” indicating that antibody levels dropped quicker and remained low for a longer period in treated compared to untreated animals (Figure 1b). The proportion of the total variation in anti-*O. ostertagi* antibody levels that resided at the herd, cow and residual level was 12, 52% and 36%, respectively.

Table 2 lists the faecal egg count results of the two studied herds. The EPG of trichostrongyle-type eggs was, on average (± standard error) 26 (±42) and 13 (±29), for the two herds, respectively. The Spearman rank correlation coefficient between FEC and anti-*O. ostertagi* antibody level of individual milk samples taken within 30 days post calving was *R* = 0.42 (*P* = 0.03).

**Table 2:** **Results of the faecal egg counts measured around calving on two dairy herds (N: the number of samples; P25 and P75: the first and third quartile, respectively)**

	**Fecal egg count results (EPG)**				
	***N***	**Median**	**P25**	**P75**	**Range**
**Herd 1**	34	14	2	28	0 - 228
**Herd 2**	49	2	0	8	0 - 128

### Overall effect of eprinomectin treatment on milk production

Analysis of the treatment response on the test day milk production records by a linear mixed model showed a significant increase of 0.97 (95% confidence interval: 0.17 to 1.77) kg milk/day per cow in eprinomectin treated animals (P < 0.02; Table 3). The model controlled for the factors lactation number, somatic cell count, number of days in milk and the year quarter in which calving occurred. The herd, cow and residual level explained 27, 35 and 38% of the total variation, respectively.

**Table 3:** **The results of a linear mixed model to estimate the effect of eprinomectin treatment around calving on daily milk production in 11 herds (based on 477 cows)**

**Variable**	**Estimate**	**Lower limit**	**Upper limit**	**t Value**	***P***
		**95% CI**	**95% CI**		
Intercept	95.80	88.66	102.93	26.36	<0.001
Eprinomectin (vs. control)	0.97	0.16	1.77	2.36	0.019
Lactation number (baseline is third lactation or higher)					<0.001
First	−7.15	−8.17	−6.11	−13.66	<0.001
Second	−3.05	−4.03	−2.07	−6.13	<0.001
Ln (SCC/1000)	−1.21	−1.36	−1.06	−16.12	<0.001
DIM	−0.07	−0.07	−0.06	−43.02	<0.001
Wilmink	−58.02	−66.05	−49.99	−14.16	<0.001
Year quarter (baseline is 4^th^ year quarter)					0.0064
First	−1.23	−2.31	−0.15	−2.23	0.026
Second	−1.48	−2.68	−0.27	−2.41	0.016
Third	−1.84	−2.94	−0.74	−3.29	0.001
**Random Effects**	**Variance**	**S.E.**	**Proportion of total variance (%)**	**Z value**	***P***
Herd	12.95	6.00	27	2.16	0.015
Animal	16.50	1.28	35	12.90	<0.001
Residual	17.92	0.44	38	40.48	<0.001

**Figure 2:**
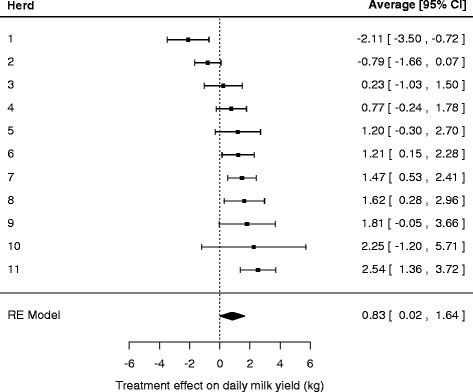
**Average response in daily milk yield after eprinomectin treatment at calving for each herd separately and the overall inverse variance weighted average computed with a random effects model.** Bars represent the 95% confidence interval.

The forest plot in Figure 2 shows the variation in treatment effects between herds. The precision-weighted average treatment effect was 0.83 (95% confidence interval: 0.02 to 1.64) kg milk/cow per day.

**Figure 3:**
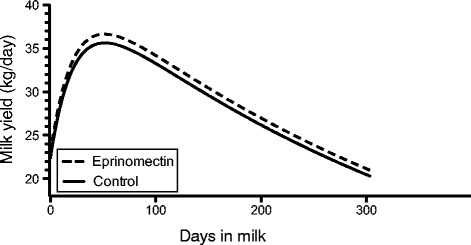
**Average lactation curve of cows treated with eprinomectin at calving versus untreated cows.**

Although the Milkbot® parameters (scale, ramp, decay) did not differ significantly between groups, the lactation curve (Figure 3) suggested that eprinomectin treatment at calving resulted in a higher peak production and that this effect was maintained throughout the entire lactation.

### The association between non-invasive indicators and the milk yield response after anthelmintic treatment

**Figure 4:**
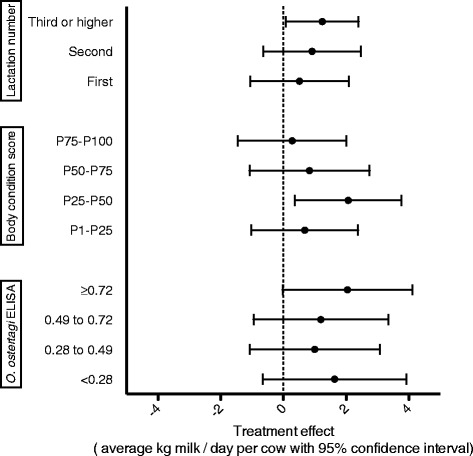
**Estimated effect of eprinomectin treatment at calving on daily milk yield (kg) per cow according to different potential selection parameters for anthelmintic treatment.** Error bars represent the 95%-confidence interval. Categories for body condition score and *O. ostertagi* ELISA are based on quartiles.

**Figure 5:**
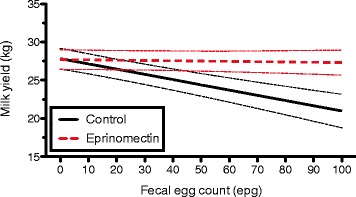
**Relationship between faecal egg count results and daily milk yield (kg) per cow measured at two herds.** The dotted lines represent the standard error of the mean.

An overview of the effect of eprinomectin treatment on daily milk yield per animal in relation to the evaluated parameters is provided in Figure 4. Animals in third lactation or higher had a significant increase in daily milk production after treatment of 1.24 (95% confidence interval: 0.08 to 2.40) kg milk/cow per day. A large and almost significant effect of 2.05 (95% confidence interval: −0.03 to 4.12) kg milk/cow per day, was also found in the cows in the 4^th^ quartile of anti-*O. ostertagi* antibody level pre-treatment (ODR ≥0.72). A significant treatment effect was observed in animals with a BCS centered to the herd mean in the second quartile. The treatment effects were similar in both seasons. Finally, on the 2 herds where FECs were performed, a significant linear association was found between treatment effect and FEC (*P* = 0.01). This relationship is illustrated in Figure 5.

## Discussion

On commercial dairy farms under European conditions, anthelmintic treatment administered shortly after calving, resulted in an average increase of 0.97 kg milk/day per cow. A recent study on anthelmintic treatment at calving in Canada found a maximal treatment effect of 0.67 kg milk/day per cow, which is considerably lower than some of the observations reported here [12]. A study performed in New Zealand, found a positive effect after treatment in only one of the three participating herds of 0.78 kg energy corrected milk/day per cow [24]. Such differences from our observations underline the need to evaluate treatment effects on milk production in different geographical locations and under different management conditions.

Although the treatment was randomly assigned, it was not blinded to the farmer which could have led to a biased treatment effect estimate. Non-blinding may result in occasional use of the anthelmintic product for animals assigned to the control group, however this was rarely recorded. At the end of the trial, the prescribed treatment assignment (‘treat’ or ‘not treat’) had not been followed for a total of 45 animals. The farmer also had to assess the appropriate dosing based on the estimated body weight, which is likely to lead to underdosing as compared to dosing under controlled conditions by the researcher. Furthermore, there is a higher risk of incorrect storage of the anthelmintic product on the farm, which would also lead to a reduced treatment effect.

Previously, we observed a significant effect of anthelmintic treatment at housing on the anti-*O. ostertagi* antibody level in bulk milk samples [7]. In that study, no re-exposure to GI nematodes in the months following treatment took place and the effect was largest 3–4 months after treatment. In the present study, we observed a significant treatment effect on the anti-*O. ostertagi* antibody level in individual milk samples. However, the anti-*O. ostertagi* antibody levels dropped in both the treated and untreated group and the differences only became clear after >6 months, when the anti-*O. ostertagi* antibody levels started to rise again. The concurrent drop in both the treated and untreated group may be explained by a dilution effect since milk production is highest 2–3 months following treatment. However, this effect has previously been estimated to be small [25]. A second possible reason is a generally lower exposure to GI nematodes in the herd caused by treatment approximate 50% of the animals.

There was a considerable variation in treatment response between the different herds. In one herd, there was a significant negative effect of treatment on milk production. Interestingly, although the animals were grazed up to the start of the trial, further investigation revealed that the animals in this herd had not been at pasture during the entire study period. This, together with the low average (± standard deviation) bulk tank milk *O. ostertagi* ELISA results (0.52 ODR (±0.09)) suggests a very low worm exposure in this herd. When this herd was excluded from the analysis, the overall treatment effect (95% confidence interval) on milk yield was 1.09 (0.42 – 1.76) kg milk per day per cow.

Currently, targeted selective treatments (TST), where anthelmintic treatments are given to selected individuals within a herd, are promoted as a method of maintaining animal performance whilst reducing the selection of anthelmintic resistant nematodes [2]. However, until now, TST have been mostly evaluated in sheep [26-31] and few data are available to determine which parameters are useful in identifying cattle that would benefit most from anthelmintic treatment. Previously the anti-*O. ostertagi* antibody level in individual milk samples has been proposed as a useful indicator of which animals would benefit most from treatment [3,11,12,16]. Here, we confirm that the highest treatment responses were observed in the cows with the highest anti-*O. ostertagi* antibody level. However, considerable responses were also observed in animals with low anti-*O. ostertagi* antibody levels, which corresponds to a recent study of Ravinet et al. [8]. This indicates that significant economic potential may be lost if only animals with a high anti-*O. ostertagi* antibody level are treated and highlights the need to assess the broader economic implications on-farm when this indicator would be used as the principal treatment criterion.

In addition to the anti-*O. ostertagi* antibody level, we identified 2 other promising candidates for targeting individual treatments: age and FEC. Although higher treatment responses in older cattle may appear counterintuitive, as these animals are considered to be functionally immune to gastrointestinal nematodes, several previous studies reported higher treatment responses with increasing cow age [3,13,25]. As discussed before [3], the latter may be attributed to a different priority of nutrient allocation between primi- and multiparous cows [13] or the higher energy requirements of the immune response to GI nematodes in older animals, as observed in sheep [32]. Finally, it may also be considered that the level of GI nematode infection in primiparous cows is generally lower than in older cows. A recent survey in Europe showed that anthelmintic control measures in young stock are relatively intensive resulting in low infection levels of first-season grazing calves and a compromised build-up of immunity against these parasites [33].

The milk production response increased in those animals with higher FECs at calving. This is surprising because FECs are generally considered as a poor indicator of the GI nematode infection level in cattle [34,35]. O’Farrell et al. [17] and Walsh et al. [36] found no relationship between milk yield response after anthelmintic treatment and FEC. However, in a recent study, a significantly lower milk production was observed in animals with a FEC >10 EPG, although whether these animals would also have a higher milk yield after anthelmintic treatment was not investigated [37]. Our results require to be confirmed because they are only based on observations in 2 herds. A possible reason why we observe this relationship is because a much more sensitive technique (FLOTAC with analytical sensitivity of 2 EPG) was used than in the previous studies.

A significant positive treatment effect on milk production was found for cows with a BCS between the 25^th^ and 50^th^ percentile. Despite our attempt to standardize this variable by centering it to the herd mean, the results for the BCS should still be interpreted with caution since important between-farmer variability may remain present and the study design did not take BCS into account as blocking parameter. Based on the described relationship between BCS around calving and subsequent milk yield [38], it could be expected that production responses are lower in cows with either a very low or very high BCS. Such a relationship was previously observed in a study investigating milk yield responses after flukicide treatments [9].

## Conclusions

In conclusion, this randomized controlled field trial demonstrates that eprinomectin treatment at calving reduces the infection levels with gastro-intestinal nematodes and increases the daily milk yield in the following lactation. Treatment responses were highest in animals in their third or higher lactation, with a high pre-treatment anti-*O. ostertagi* antibody level in a milk sample of the previous lactation and in animals with a moderate body condition score. Further research needs to be done to assess the economic impact of selective treatment approaches, which greatly influences the eventual uptake of these indicators in practice.
